# Challenges and Prospects of Photocatalytic Applications Utilizing Semiconductor Nanocrystals

**DOI:** 10.3389/fchem.2018.00353

**Published:** 2018-08-15

**Authors:** Pavel Moroz, Anthony Boddy, Mikhail Zamkov

**Affiliations:** ^1^The Center for Photochemical Sciences, Bowling Green State University Bowling Green, OH, United States; ^2^Department of Physics and Astronomy, Bowling Green State University, Bowling Green, OH, United States; ^3^Department of Biological Sciences, Bowling Green State University, Bowling Green, OH, United States

**Keywords:** photocatalysis, nanocrystals (NC), exciton dynamics, heterostructures, triplet sensitization

## Abstract

Photocatalytic systems based on colloidal semiconductor nanocrystals have gained considerable attention owing to potential benefits that include a visible-range light extinction and a low spatial overlap of photoinduced charges. When coupled to metal catalysts, nanocrystal sensitizers have demonstrated a compelling performance in homogenous photoreduction reactions, including the degradation of organic dyes and hydrogen generation. Going beyond half-cycle reactions, however, the progress in the field of nanocrystal photocatalysis has been rather limited. Here, we review some of the challenges associated with photocatalytic applications of colloidal semiconductor nanocrystals and highlight possible directions aimed toward their resolution. A particular emphasis was made on new paradigms in this field, including the possibility of harvesting triplet excitons and utilizing nanocrystal assemblies to accumulate multiple charges at the reaction site.

The prospect of employing colloidal semiconductor nanocrystals (NCs) in photocatalytic applications is inspired by unique advantages of quantum confined semiconductors over more traditional systems based on transition metal oxides (Schultz and Yoon, 2014) and precious metal coordination compounds (Concepcion et al., 2009). The benefit of inorganic nanocrystal catalysts lies in the combination of tunable redox energies and a large density of states across the visible spectrum, which gives rise to the energetic feasibility for overall water splitting (e.g., CdS, CdSe). While the photooxidation of water by semiconductor colloids has not yet been realized at a meaningful performance level, the photoreduction half-cycle reactions were shown to reach turnover numbers (TONs) in excess of 10^5^, which was attributed to an efficient charge separation between the semiconductor domain and an appended metal catalyst [Ni (Simon et al., 2014; Chai et al., 2016), Pt (Bao et al., 2008; Berr et al., 2010), Pd (Raza et al., 2017), or Au (Costi et al., 2008)]. Such metal-semiconductor assemblies were also deemed cost effective as most semiconductor colloids contained no precious metals with several architectures featuring heavy metal-free compositions [CuInS_2_ (Zhou et al., 2017), Cu_2_ZnSnS_4_ (Yu et al., 2014a), CuIn_1−x_Ga_x_S_2_ (Yu et al., 2014b) NCs].

One of the early successful demonstrations of nanocrystal-based photocatalytic systems represents a heterostructured combination of a CdS nanocrystal sensitizer coupled to a Pt reduction co-catalyst. This architecture was shown to be up to 60% efficient in catalyzing the sacrificial reduction of protons (Costi et al., 2008; Zhou et al., 2017), and organic molecules (Brown et al., 2016; Jensen et al., 2016) under visible radiation (λ ≈ 450 nm).

A compelling performance of the CdS/Pt assembly was attributed to the two key aspects of this hybrid architecture: (i) the fast removal of photocorrosive holes from the CdS domain (Acharya et al., 2011), aided by electron-donating surface ligands, and (ii) a significant driving force for the electron injection into the metal catalyst (Khon et al., 2011). Further increases in the proton reduction quantum yield were made possible by employing heterostructured CdSe/CdS and ZnSe/CdS nano-interfaces within the sensitizer component (Zhu et al., 2012; Kalisman et al., 2016), which allowed increasing the spatial extent of the photoinduced charge separation between hole-rich (CdSe, ZnSe) and electron-rich (Pt) domains (see Figures 1a–c; Hewa-Kasakarage et al., 2009; O'Connor et al., 2012; Kalisman et al., 2016).

**Figure 1:**
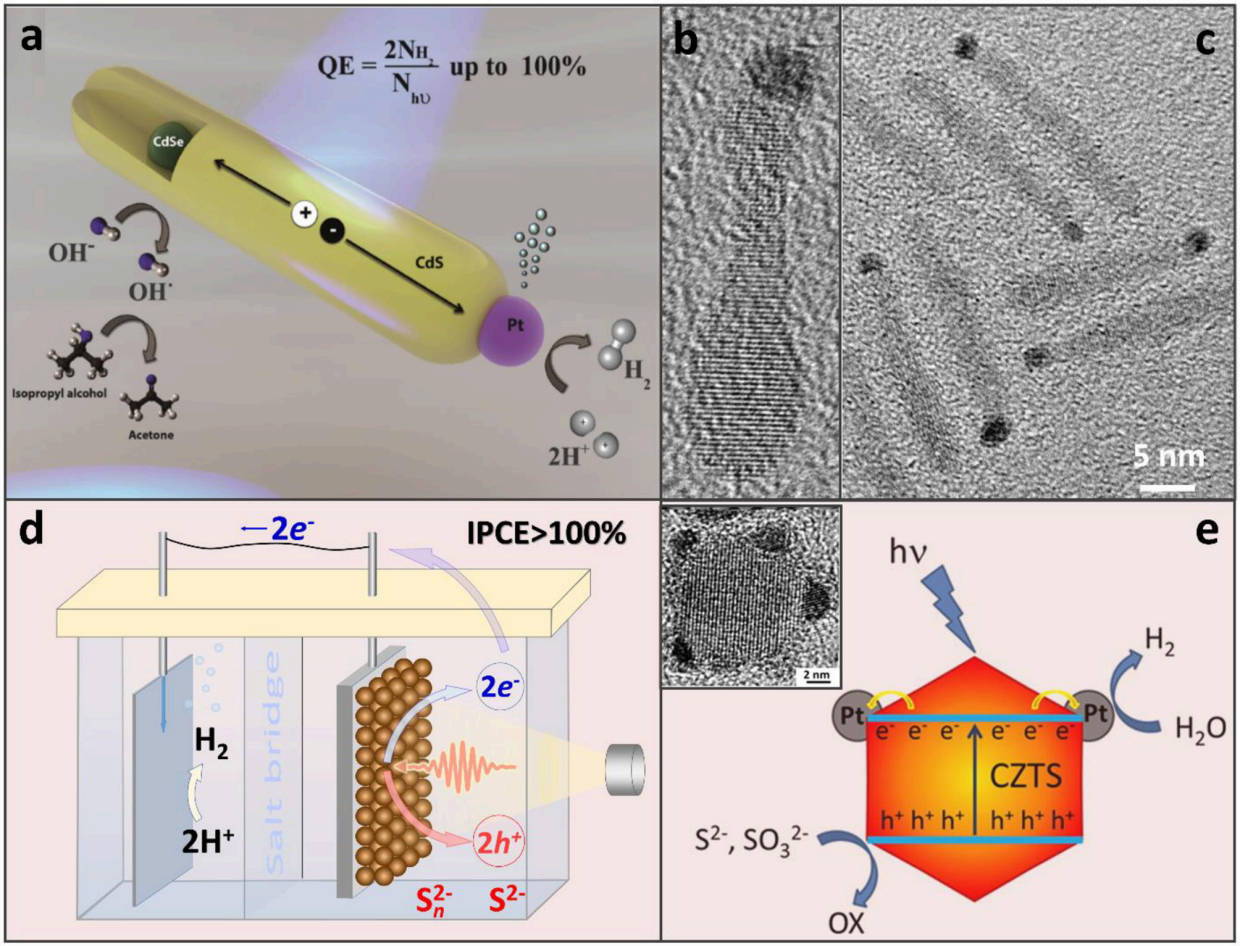
**(a)** CdSe/CdS nanorods appended with a Pt co-catalyst that serves the role of an electron sink for the accumulation of photoinduced electrons at a single reductive site. Adapted with permission from Kalisman et al. (2016). Copyright 2016 American Chemical Society. **(b,c)** TEM images of CdSe/CdS nanorods selectively tipped with a Pt catalyst on one side. Based on the location of the CdSe bulb, Pt appears to grow on an opposite end. Adapted with permission from Khon et al. (2013). Copyright 2016 American Chemical Society. **(d)** Photoelectrochemical hydrogen evolution from aqueous Na_2_S solution with over 100% of incident photon-to-current efficiency (IPCE). PbS NC-based active region of the working electrode (right, brown spheres) where one photon may be converted into two electron-hole pairs via MEG (Yang et al., 2014). Energized electrons are then transferred to the dark electrode (left) for H_2_ production and sulfide ions are oxidized by the holes. **(d)** Illustration of the H_2_ evolution on CZTS-Pt heterostructured nanoparticles in the presence of S^2−^ and SO${}_{3}^{2-}$ hole scavengers. Adapted with permission from Yu et al. (2014a).

While Pt remains to be the most efficient co-catalyst, other compounds have also been explored in combination with semiconductor colloids to drive sacrificial reduction reactions. For instance, Ni-based heterostructures comprising a CdS sensitizer have enabled up to 50% of the H_2_ production internal efficiency (Simon et al., 2014; Zhukovskyi et al., 2015). Non-noble metal co-catalysts, including Ni_2_P, Co_2_P, FePt metal phosphides, and bimetallic compounds appended to CdS nanocrystals, have also been investigated as an alternative to Pt (Cao et al., 2014, 2015; Cheng et al., 2016). Regarding the sensitizer component, attempts to reduce the Cd content have led to its partial replacement by Cu in Cu_1.94_S–Zn_x_Cd_1−x_S heteronanorods (Chen et al., 2016), or a complete removal of Cd in CZTS/Pt heterostructures (see Figure 1e). Photocatalytic applications of semiconductor nanocrystal assemblies have been attempted as well (Warren et al., 2013; Yang et al., 2014). As an interesting example of an emerging paradigm, photoelectrodes comprising PbS semiconductor nanocrystal solids were shown to generate multiple photoinduced electrons-hole pairs from a single UV exciton, a phenomenon known as the multiple exciton generation (MEG) (Yan et al., 2017; Zamkov, 2017). A particular advantage of the MEG effect in photoelectrochemical cells over the same phenomenon in photovoltaic devices (Beard et al., 2013) was the reverse anode architecture that did not filter the excitation light through the oxide hole-blocking layer (Yan et al., 2017).

Many years of extensive research on photocatalytic applications of semiconductor nanocrystals has identified the key performance-limiting factors of these systems, most of which could be traced to the instability of inorganic semiconductors under catalytic conditions. Depending on a particular material, the catalytic performance of corresponding nanocrystals was shown to suffer from such issues as photocorrosion, a short excited state lifetime, poor colloidal stability in acidic solutions, or a limited ability to convey the photoinduced charges to active catalytic sites.

Photocorrosion of the semiconductor material due to the accumulation of photoinduced holes represents one of the most significant issues impeding photocatalytic applications of colloidal nanocrystals. Since chalcogenides are readily oxidized (Kamat et al., 2014), nanocrystal catalysts comprising CdS, CdSe, CZTS, or PbS semiconductors eventually undergo some degree of the chemical degradation, particularly when positive charges are not regenerated quickly. Even if the timely regeneration of holes is achieved via sacrificial agents, the photooxidizing energy could still be transferred to surface ligands triggering their desorption and the subsequent nanoparticle aggregation (Acharya et al., 2011; Hines and Kamat, 2014). For instance, commonly used mercaptopropionic (MPA) or mercaptoundecanoic (MUA) acids ligands are readily oxidized by scavenged holes resulting in the formation of disulfides. Consequently, these thiolates need to be continuously replaced in order to sustain the reduction half-cycle rate. One effective strategy is to use an abundant concentration of the scavenger moiety in solution (e.g., ascorbic acid) (Han et al., 2012) that quickly relieves the nanocrystal-ligand system of a positive charge.

Semiconductor photocorrosion could also be the result of defective surfaces that tend to localize positive charges at potential energy minima (Utterback et al., 2016). It was shown that holes diffuse through such defects by hopping, thus creating hot spots for chalcogenide oxidation and other side reactions. The localization of holes inside the nanocrystal sensitizer was also shown to reduce the ensuing catalytic activity due to a low sacrificial regeneration rate (Utterback et al., 2016). For instance, such a confinement of holes may be responsible for a relatively low photocatalytic activity of CuInS_2_ NCs where these charges become trapped on Cu^+^ ions within the lattice structure (Leach and Macdonald, 2016; Fuhr et al., 2017).

In addition to photocorrosion, the performance of nanocrystal-based photocatalytic systems could, in some cases, be limited by short lifetimes of singlet excitons. Indeed, homogenous systems utilizing organometallic dyes benefit from an effective way of storing the photon energy through a rapid intersystem crossing into a triplet state (McCusker and Castellano, 2016; Twilton et al., 2017), which lifetime can extend into hundreds of microseconds [e.g., the triplet state of Ir(ppy)_3_] (Hofbeck and Yersin, 2010). The radiative lifetimes of singlet excitons in cadmium chalcogenide nanocrystals, on the other hand, are in the 10–100 ns range, which requires the dissociation of excitons on a faster time scale. In practice, the temporal window for a photoinduced charge transfer to a catalyst is even shorter due to competing pathways of non-radiative exciton dissociation at trap states, caused by the increased density of dangling bonds in aqueous environments.

The task of increasing excited state lifetimes of colloidal nanocrystals could be accomplished both by extending radiative lifetimes and reducing the density of charge-localizing trap states. In regard to the former condition, some groups have employed a heterojunction of the two semiconductor materials exhibiting a type II band edge alignment at the interface, which increases the spatial separation of photoinduced charges (Amirav and Alivisatos, 2010). The benefits of this strategy were demonstrated through the observation of a nearly 100% quantum yield (QY) for MV^2+^ photo reduction in Pt-tipped CdSe/CdS nanorods (Zhu et al., 2012) vs. a 60% QY observed for in Pt-tipped CdS structures (Bao et al., 2008). In addition to CdSe/CdS semiconductor combination, type II heterojunctions utilizing ZnSe/CdS (O'Connor et al., 2012) and Cu_1.94_S–Zn_x_/Cd_1−x_S (Chen et al., 2016) semiconductors have also been shown to enhance excited state lifetimes beyond those of single-phase nanocrystals. One potential issue with employing such type II interfaces in photocatalytic applications concerns the fact that one of the separated charges resides in the enclosed domain, which is shielded from the external environment by the other material (e.g., a core/shell or dot-in-a-rod geometry) (Perera et al., 2012). As a result, the confined carrier cannot be efficiently regenerated. In order to expose both semiconductor domains of a type II heterostructure to a redox environment, chemical etching could be employed. It was shown that etching of spatially-asymmetric CdSe/CdS nanorods results in the formation dimer-like structures where both donor and acceptor components are in direct contact with the external environment, resulting in the increased catalytic activity (Khon et al., 2013).

One emerging strategy for enhancing radiative lifetimes of excitons in nanocrystal-based photocatalytic systems relies on doping of semiconductors with transition metal ions. Lattice incorporated Mn^2+^ or Cu^+^ can serve as hole trap sites promoting exciton localization in the bulk of the nanocrystal. In colloidal solutions, this strategy can allow extending excited state lifetimes into a microsecond range for Ag^+^- and Cu^+^- doped CdSe NCs (Kholmicheva et al., 2017; Nelson et al., 2017), or even a millisecond range for Mn^2+^-doped ZnSe/ZnS core/shell (Pu et al., 2016) and CdS QDs (Knowles et al., 2015). The photocatalytic applications of doped semiconductor nanocrystals, however, have not yet been explored. A potentially adverse aspect of this architecture concerns the slow regeneration of photoinduced holes localized at dopant sites. Another possible strategy for increasing the nanocrystal excited state lifetime is based on reducing the density of surface traps. The two approaches that were shown successful in this regard have employed either exciton-delocalizing ligands or a defect-passivating semiconductor shell (Grenland et al., 2017). The former strategy was recently demonstrated through the employment of hole-accepting ligand molecules that were covalently linked to nanocrystals *via* a thiolate binding group (Ding et al., 2015; Olshansky et al., 2015). By using ferrocene ligands with different alkyl chain lengths it was possible to find an optimal driving force for hole removal. As a result, existing surface traps could be mitigated without compromising the ability to extract photoinduced charges. Similarly, surface defects can be neutralized through the use of exciton delocalizing PZT ligands that were shown to scavenge holes on a picosecond to nanosecond time scale in CdS and CdSe NCs (Wu et al., 2015; Lian et al., 2016).

An important prerequisite of any photocatalytic system is the ability to accumulate photoinduced charges at an active site. This is particularly relevant in the case of multi-electron reactions where charges need to be collected onto a single catalytic domain. In regard to semiconductor sensitizers, this aspect was experimentally confirmed through the observation of a diminishing hydrogen production efficiency in Pt-decorated CdSe@CdS rods with the increasing number of appended Pt catalysts (Nakibli et al., 2015). In particular, nanorods tipped with a single Pt domain showed ~1.6 times the efficiency for the H_2_ production as compared to nanorods containing two Pt domains. This result was attributed to the competition of the two metal tips for photoinduced electrons absorbed by a shared semiconductor domain. The importance of funneling the absorbed energy to a catalytic site was also illustrated by an earlier study (Amirav and Alivisatos, 2010) showing an increased H_2_ production rate by Pt-tipped CdS nanorods with an increasing CdS length.

The above mentioned benefits and drawbacks of nanocrystal-based photocatalytic systems, identified and investigated by the community over the course of many years, allow formulating design principles for the future development in this field. The key challenges to be addressed include the suppression of photocorrosion by photoinduced holes, the reduction of the trap states' effect, maintaining a suitable pH balance to prevent aggregation in acidic buffers, and funneling the excitation energy to an active reaction site (Aldana et al., 2005). Below we would like to discuss the design of the two emerging nanocrystal photocatalytic platforms that show strong potential for overcoming the aforementioned challenges. The first is based on a recently demonstrated ability of semiconductor nanocrystals to harvest triplet excitons through the Dexter energy transfer to coordination compounds exhibiting long-lived excited states. The second approach utilizes closed-coupled nanocrystal assemblies to funnel the absorbed energy to a catalytic site. Below we described the two paradigms in more detail.

The demonstrated ability of semiconductor nanocrystals to harvest triplet excitons (Mongin et al., 2016) offers new opportunities in light sensitization of photoinduced redox reactions (Figure 2a). By engaging in the Dexter energy transfer with molecular photoredox catalysts, such as [Ru(bpy)_3_]^2+^ or Ir(ppy)_3_ coordination compounds (Arias-Rotondo and McCusker, 2016), nanocrystal energy could be transformed into a long lived state with minimal energy losses. While such energy transfer has been demonstrated for simple molecules, such as ruberene (Wu et al., 2016) or 9-anthracenecarboxylic acid (ACA) (Mongin et al., 2016), sensitizations of triplet excited states of photoredox coordination compounds is imminent. Of a particular interest are metal polypyridyl complexes, which exhibit excellent oxidizing and reducing properties. For instance photoexcited [Ru(bpy)_3_]^3+^ can oxidize water into O_2_ and protons *via* a metal oxide catalyst (Hara et al., 2000), while, [Ru(bpy)_3_]^2+*^ triplet states can be utilized for reducing methylviologen (via ligands), a recyclable carrier of electrons.

**Figure 2:**
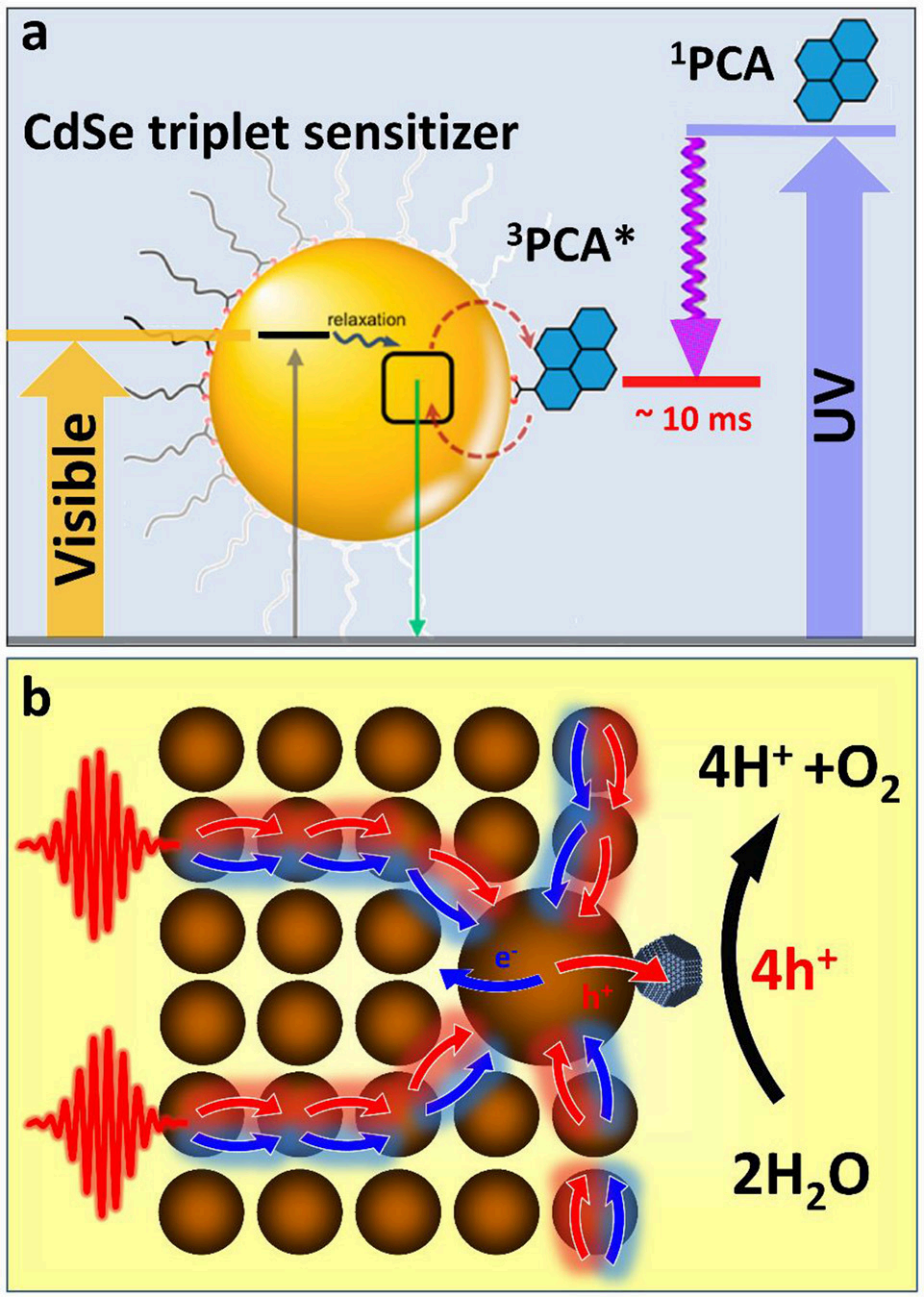
**(a)** Triplet sensitization of 1-pyrenecarboxylic acid (PCA) molecular acceptor by CdSe NCs via photoinduced triplet–triplet energy transfer (TTET). Close to 90% of photons absorbed by the semiconductor QD could be stored in form of long-lived triplet states of ^3^PCA*. The associated exciton energy loss resulting from TTET is less than 20%, which compares favorably with a >50% energy loss accompanying ^1^PCA → ^3^PCA intersystem crossing. Adapted with permission from Garakyaraghi and Castellano (2018). Copyright 2016 American Chemical Society. **(b)** A proposed scheme for funneling the photoinduced energy in assemblies of semiconductor nanocrystals via the diffusion toward the low-energy gap reaction center. This strategy benefits multi-electron catalytic processes by increasing the probability of multiple charges to be collected on the same catalytic site.

Employing semiconductor nanocrystals as triplet sensitizers of photoredox coordination compounds would allow avoiding many aforementioned issues of nanocrystal photocatalytic materials related to photocorrosion, hole regeneration, and short singlet lifetime. On the other hand, coupling nanocrystal sensitizers to organometallic catalysts will extend the usable portion of the solar spectrum into the visible range. This is because the excitation of a triplet state undergoes *via* a photon absorption into a singlet metal-ligand charge transfer state (^1^MLCT) followed by a rapid intersystem crossing to a ^3^MLCT state, which is commonly accompanied by an ~1 eV energy loss (due to large splitting of singlet and triplet states). Since such singlet-triplet splitting in semiconductor nanocrystals is usually much smaller (within thermal kT ~ 30 meV), the associated energy loss will be reduced. Furthermore, a molar absorptivity of CdSe at 400–450 nm is 20 times greater (Yu et al., 2003) than that of the ^1^MLCT transition in [Ru(bpy)_3_]^2+^ (~13,000 M^−1^ cm^−1^in acetonitrile). Considering that the efficiency of triplet exciton transfer from CdSe to organic acceptors, such as ACA is over 90% (Mongin et al., 2016), there is an expected benefit in employing semiconductor nanocrystals for sensitizing redox reactions.

Like any excitonic system, an assembly of semiconductor nanocrystals can be designed to transfer the photoinduced energy from the excitation site toward the potential energy minima through the process of exciton diffusion. Such funneling of the photoinduced energy is often utilized by biological systems as a mechanism to drive multiple carriers to the reaction center where multielectron catalytic reactions can subsequently take place. For instance during the oxygenic photosynthesis in plants, light is absorbed by hundreds of pigments (e.g., chlorophylls) that transfer the photoinduced energy to a small number of special pigments (P680), which are capable of charge separation (Blankenship, 2013). P680 will then share a photoinduced hole with a water-oxidizing complex (WOC) (Najafpour et al., 2017). After four oxidizing equivalents have been stored at the WOC site, it obtains four electrons from water molecules causing H_2_O splitting. We expect that nanocrystal assemblies could be employed in a similar manner in order to drive multielectron catalytic processes, such as water oxidation or hydrogen production. For instance, the diffusion of excitons in a nanocrystal solid to a nanoparticle with the smallest band gap (Kholmicheva et al., 2015) (an equivalent of the P680 pigment in PSII) can be used to collect multiple excitons in the same spot. The accepting dot could be appended with a catalyst that would assist the charge separation to store photoinduced charges. The presence of an electron- (or hole-) accepting catalysis would also allow avoiding the multiexciton populations on a single nanocrystal, which are subject to a rapid decay through Auger recombination. Such nanocrystal assembly could be incorporated into a photoelectrochemical cell (see Figure 1d) or even harnessed within an “artificial leaf” platform (Liu et al., 2016). The key advantage of this architecture lies in the sequential collection of multiple charges within the same catalytic complex (Figure 2b). Zero-dimensional nanocrystals in these assemblies could be substituted with either one- or two-dimensional nanostructures in order to increase the energy transfer efficiency and reduce Auger recombination rates. Notably, without such an energy “antenna,” nanocrystal-based water splitting systems would exhibit very low efficiencies (Kalisman et al., 2015) even when designed with a robust corrosion suppression mechanism.

In summary, the prospect of employing semiconductor nanocrystals in photocatalysis offers a number of unique benefits related to a spatially-extended charge separation and visible-range light absorption, which have been confirmed through a compelling performance in reduction half-reactions. In order for such systems to become practical, however, additional challenges need to be resolved. These pertain to the semiconductor photocorrosion, short excited state lifetimes, and poor control over energy transfer to catalytic sites. To resolve these issues, several emerging strategies have been proposed and discussed. Among potential solutions, harnessing nanocrystals as triplet sensitizers of photoredox coordination compounds is expected to enhance the absorption characteristics of the latter while decreasing the damage of the semiconductor. Assemblies of inorganic colloids can also be used for funneling the photoinduced energy to reactive sites in a manner analogs to the action of chlorophylls in PSII. This geometry could inspire a cascade like design of photosynthetic assemblies for water oxidation.

## Author contributions

All authors have contributed equally to preparing the review article.

### Conflict of interest statement

The authors declare that the research was conducted in the absence of any commercial or financial relationships that could be construed as a potential conflict of interest.

## References

[B1] Acharya K. P. Khnayzer R. S. O'Connor T. Diederich G. Kirsanova M. Klinkova A.. (2011). The role of hole localization in sacrificial hydrogen production by semiconductor–metal heterostructured nanocrystals. Nano Lett. 11, 2919–2926. 10.1021/nl201388c 21615085

[B2] Aldana J. Lavelle N. Wang Y. Peng X. (2005). Size-dependent dissociation pH of thiolate ligands from cadmium chalcogenide nanocrystals. J. Am. Chem. Soc. 127, 2496–2504. 10.1021/ja047000+ 15725004

[B3] Amirav L. Alivisatos A. P. (2010). Photocatalytic hydrogen production with tunable nanorod heterostructures. J. Phys. Chem. Lett. 1, 1051–1054. 10.1021/jz100075c

[B4] Arias-Rotondo D. M. McCusker J. K. (2016). The photophysics of photoredox catalysis: a roadmap for catalyst design. Chem. Soc. Rev. 45, 5803–5820. 10.1039/C6CS00526H 27711624

[B5] Bao N. Shen L. Takata T. Domen K. (2008). Self-templated synthesis of nanoporous CdS nanostructures for highly efficient photocatalytic hydrogen production under visible light. Chem. Mater. 20, 110–117. 10.1021/cm7029344

[B6] Beard M. C. Luther J. M. Semonin O. E. Nozik A. J. (2013). Third generation photovoltaics based on multiple exciton generation in quantum confined semiconductors. Acc. Chem. Res. 46, 1252–1260. 10.1021/ar3001958 23113604

[B7] Berr M. Vaneski A. Susha A. S. Rodríguez-Fernández J. Döblinger M. Jäckel F. (2010). Colloidal CdS nanorods decorated with subnanometer sized Pt clusters for photocatalytic hydrogen generation. Appl. Phys. Lett. 97:093108 10.1063/1.3480613

[B8] Blankenship R. E. (2013). Molecular Mechanisms of Photosynthesis. Malden, MA; Oxford: Blackwell Science.

[B9] Brown K. A. Harris D. F. Wilker M. B. Rasmussen A. Khadka N. Hamby H.. (2016). Light-driven dinitrogen reduction catalyzed by a CdS:nitrogenase MoFe protein biohybrid. Science 352, 448–450. 10.1126/science.aaf2091 27102481

[B10] Cao S. Chen Y. Hou C. C. Lv X. J. Fu W. F. (2015). Cobalt phosphide as a highly active non-precious metal cocatalyst for photocatalytic hydrogen production under visible light irradiation. J. Mater. Chem. A 3:6096–6101. 10.1039/C4TA07149B

[B11] Cao S. Chen Y. Wang C. J. He P. Fu W. F. (2014). Highly efficient photocatalytic hydrogen evolution by nickel phosphide nanoparticles from aqueous solution. Chem. Commun. 50, 10427–10429. 10.1039/C4CC05026F 25059389

[B12] Chai Z. Zeng T. T. Li Q. Lu L. Q. Xiao W. J. Xu D. (2016). Efficient visible light-driven splitting of alcohols into hydrogen and corresponding carbonyl compounds over a Ni-modified CdS photocatalyst. J. Am. Chem. Soc. 138, 10128–10131. 10.1021/jacs.6b06860 27477237

[B13] Chen Y. Zhao S. Wang X. Peng Q. Lin R. Wang Y.. (2016). Synergetic integration of Cu_1.94_S–Zn_x_Cd_1−x_S heteronanorods for enhanced visible-light-driven photocatalytic hydrogen production. J. Am. Chem. Soc. 138, 4286–4289. 10.1021/jacs.5b12666 26998730

[B14] Cheng H. Lv X. J. Cao S. Zhao Z. Y. Chen Y. Fu W. F. (2016). Robustly photogenerating H_2_ in water using FeP/CdS catalyst under solar irradiation. Sci. Rep. 6:19846. 10.1038/srep19846 26818001 PMC4730145

[B15] Concepcion J. J. Jurss J. W. Brennaman M. K. Hoertz P. G. Patrocinio A. O. Iha N. Y. M.. (2009). Making oxygen with ruthenium complexes. Acc. Chem. Res. 42, 1954–1965. 10.1021/ar9001526 19817345

[B16] Costi R. Saunders A. E. Elmalem E. Salant A. Banin U. (2008). Visible light-induced charge retention and photocatalysis with hybrid CdSe–Au nanodumbbells. Nano Lett. 8, 637–641. 10.1021/nl0730514 18197720

[B17] Ding T. X. Olshansky J. H. Leone S. R. Alivisatos A. P. (2015). Efficiency of hole transfer from photoexcited quantum dots to covalently linked molecular species. J. Am. Chem. Soc. 137, 2021–2029. 10.1021/ja512278a 25591013

[B18] Fuhr A. S. Yun H. J. Makarov N. S. Li H. McDaniel H. Klimov V. I. (2017). Light emission mechanisms in CuInS_2_ quantum dots evaluated by spectral electrochemistry. ACS Photonics 4, 2425–2435. 10.1021/acsphotonics.7b00560

[B19] Garakyaraghi S. Castellano F. N. (2018). Nanocrystals for triplet sensitization: molecular behavior from quantum-confined materials. Inorg. Chem. 57, 2351–2359. 10.1021/acs.inorgchem.7b03219 29424532

[B20] Grenland J. J. Maddux C. J. A. Kelley D. F. Kelley A. M. (2017). Charge trapping versus exciton delocalization in CdSe quantum dots. J. Phys. Chem. Lett. 8, 5113–5118. 10.1021/acs.jpclett.7b02242 28972776

[B21] Han Z. Qiu F. Eisenberg R. Holland P. L. Krauss T. D. (2012). Robust photogeneration of H_2_ in water using semiconductor nanocrystals and a nickel catalyst. Science 338, 1321–1324. 10.1126/science.1227775 23138979

[B22] Hara M. Waraksa C. C. Lean J. T. Lewis B. A. Mallouk T. E. (2000). Photocatalytic water oxidation in a buffered tris(2,2'-bipyridyl)ruthenium complex-colloidal IrO_2_ system. J. Phys. Chem. A 104, 5275–5280. 10.1021/jp000321x

[B23] Hewa-Kasakarage N. N. Kirsanova M. Nemchinov A. Schmall N. El-Khoury P. Z. Tarnovsky A. N. (2009). Radiative recombination of spatially extended excitons in (ZnSe/CdS)/CdS heterostructured nanorods. J. Am. Chem. Soc. 131, 1328–1334. 10.1021/ja8082895 19119809

[B24] Hines D. A. Kamat P. V. (2014). Recent advances in quantum dot surface chemistry. ACS Appl. Mater. Interfaces 6, 3041–3057. 10.1021/am405196u 24506801

[B25] Hofbeck T. Yersin H. (2010). The triplet state of fac-Ir(ppy)_3_. Inorg. Chem. 49, 9290–9299. 10.1021/ic100872w 20853860

[B26] Jensen S. C. Bettis Homan S. Weiss E. A. (2016). Photocatalytic conversion of nitrobenzene to aniline through sequential proton-coupled one-electron transfers from a cadmium sulfide quantum dot. J. Am. Chem. Soc. 138, 1591–1600. 10.1021/jacs.5b11353 26784531

[B27] Kalisman P. Kauffmann Y. Amirav L. (2015). Photochemical oxidation on nanorod photocatalysts. J. Mater. Chem. A 7, 3261–3265. 10.1039/C4TA06164K

[B28] Kalisman P. Nakibli Y. Amirav L. (2016). Perfect photon-to- hydrogen conversion efficiency. Nano Lett. 16, 1776–1781. 10.1021/acs.nanolett.5b04813 26788824

[B29] Kamat P. V. Christians J. A. Radich J. G. (2014). Quantum dot solar cells: hole transfer as a limiting factor in boosting the photoconversion efficiency. Langmuir 30, 5716–5725. 10.1021/la500555w 24669885

[B30] Kholmicheva N. Moroz P. Bastola E. Razgoniaeva N. Bocanegra J. Shaughnessy M.. (2015). Mapping the exciton diffusion in semiconductor nanocrystal solids. ACS Nano 9, 2926–2937. 10.1021/nn507322y 25682881

[B31] Kholmicheva N. Razgoniaeva N. Yadav P. Lahey A. Erickson C. Moroz P. (2017). Enhanced emission of nanocrystal solids featuring slowly diffusive excitons. J. Phys. Chem. C 121, 1477–1487. 10.1021/acs.jpcc.6b10994

[B32] Khon E. Lambright K. Khnayzer R. S. Moroz P. Perera D. N. Butaeva E.. (2013). Improving the catalytic activity of semiconductor nanocrystals through selective domain etching. Nano Lett. 13, 2016–2023. 10.1021/nl400715n 23541120

[B33] Khon E. Mereshchenko A. Tarnovsky A. N. Acharya K. Klinkova A. Hewa-Kasakarage N. N.. (2011). Suppression of the plasmon resonance in Au/CdS colloidal nanocomposites. Nano Lett. 11, 1792–1799. 10.1021/nl200409x 21417253

[B34] Knowles K. E. Nelson H. D. Kilburn T. B. Gamelin D. R. (2015). Singlet–triplet splittings in the luminescent excited states of colloidal Cu^+^:CdSe, Cu^+^:InP, and CuInS_2_ nanocrystals: charge transfer configurations and self-trapped excitons. J. Am. Chem. Soc. 137, 13138–13147. 10.1021/jacs.5b08547 26389577

[B35] Leach A. D. P. Macdonald J. E. (2016). Optoelectronic properties of CuInS_2_ nanocrystals and their origin. J. Phys. Chem. Lett. 7, 572–583. 10.1021/acs.jpclett.5b02211 26758860

[B36] Lian S. Weinberg D. J. Harris R. D. Mohamad S. Kodaimati M. S. Weiss E. A. (2016). Subpicosecond photoinduced hole transfer from a CdS quantum dot to a molecular acceptor bound through an exciton-delocalizing ligand. ACS Nano 10, 6372–6382. 10.1021/acsnano.6b02814 27281685

[B37] Liu C. Colon B. C. Ziesack M. Silver P. A. Nocera D. G. (2016). Water splitting–biosynthetic system with CO_2_ reduction efficiencies exceeding photosynthesis. Science 352, 1210–1213. 10.1126/science.aaf5039 27257255

[B38] McCusker C. E. Castellano F. N. (2016). Materials integrating photochemical upconversion. Top. Curr. Chem. 374, 1–25. 10.1007/s41061-016-0021-7 27573144

[B39] Mongin C. Garakyaraghi S. Razgoniaeva N. Zamkov M. Castellano F. N. (2016). Direct observation of triplet energy transfer from semiconductor nanocrystals. Science 351, 369–372. 10.1126/science.aad6378 26798011

[B40] Najafpour M. M. Heidari S. Balaghi S. E. Hołynska M. Sadr M. H. Soltani B.. (2017). Proposed mechanisms for water oxidation by Photosystem II and nanosized manganese oxides. Biochim. Biophys. Acta Bioenerg. 1858, 156–174. 10.1016/j.bbabio.2016.11.007 27838231

[B41] Nakibli Y. Kalisman P. Amirav L. (2015). Less is more: the case of metal cocatalysts. J. Phys. Chem. Lett. 6, 2265–2268. 10.1021/acs.jpclett.5b00872 26266602

[B42] Nelson H. D. Hinterding S. O. M. Fainblat R. Creutz S. E. Li X. Gamelin D. R. (2017). Mid-gap states and normal vs inverted bonding in luminescent Cu^+^- and Ag^+^-doped CdSe nanocrystals. J. Am. Chem. Soc. 139, 6411–6421. 10.1021/jacs.7b01924 28421742

[B43] O'Connor T. Panov M. S. Mereshchenko A. Tarnovsky A. N. Lorek R. Perera D.. (2012). The effect of the charge-separating interface on exciton dynamics in photocatalytic colloidal heteronanocrystals. ACS Nano 6, 8156–8165. 10.1021/nn302810y 22881284

[B44] Olshansky J. H. Ding T. X. Lee Y. V. Leone S. R. Alivisatos A. P. (2015). Hole transfer from photoexcited quantum dots: the relationship between driving force and rate. J. Am. Chem. Soc. 137, 15567–15575. 10.1021/jacs.5b10856 26597761

[B45] Perera D. Lorek R. Khnayzer R. S. Moroz P. O'Connor T. Khon D. (2012). Photocatalytic activity of core/shell semiconductor nanocrystals featuring spatial separation of charges. J. Phys. Chem. C 116, 22786–22793. 10.1021/jp308921s

[B46] Pu C. Ma J. Qin H. Yan M. Fu T. Niu Y.. (2016). Doped semiconductor-nanocrystal emitters with optimal photoluminescence decay dynamics in microsecond to millisecond range: synthesis and applications. ACS Cent. Sci. 2, 32–39. 10.1021/acscentsci.5b00327 27163024 PMC4827566

[B47] Raza F. Yim D. Park J. H. Kim H. I. Jeon S. J. Kim J. H. (2017). Structuring Pd nanoparticles on 2H-WS2 nanosheets induces excellent photocatalytic activity for cross-coupling reactions under visible light. J. Am. Chem. Soc. 139, 14767–14774. 10.1021/jacs.7b08619 28953384

[B48] Schultz D. M. Yoon T. P. (2014). Solar synthesis: prospects in visible light photocatalysis. Science 343:1239176. 10.1126/science.1239176 24578578 PMC4547527

[B49] Simon T. Bouchonville N. Berr M. J. Vaneski A. Adrović A. Volbers D.. (2014). Redox shuttle mechanism enhances photocatalytic H2 generation on Ni-decorated CdS nanorods. Nat. Mater 13, 1013–1018. 10.1038/nmat4049 25087066

[B50] Twilton J. Le C. Zhang P. Shaw M. H. Evans R. W. MacMillan D. W. C. (2017). The merger of transition metal and photocatalysis. Nat. Rev. Chem. 1:0052 10.1038/s41570-017-0052

[B51] Utterback J. K. Grennell A. N. Wilker M. B. Pearce O. Eaves J. D. Dukovic G. (2016). Observation of trapped-hole diffusion on the surfaces of CdS nanorods. Nat. Chem. 8, 1061–1066. 10.1038/nchem.2566 27768112

[B52] Warren S. C. Voïtchovsky K. Dotan H. Leroy C. M. Cornuz M. Stellacci F.. (2013). Identifying champion nanostructures for solar water-splitting. Nat. Mater. 12, 842–849. 10.1038/nmat3684 23832125

[B53] Wu K. Du Y. Tang H. Chen Z. Lian T. (2015). Efficient extraction of trapped holes from colloidal CdS nanorods. J. Am. Chem. Soc. 137, 10224–10230. 10.1021/jacs.5b04564 26221916

[B54] Wu M. Congreve D. N. Wilson M. W. B. Jean J. Geva N. Welborn M. (2016). Solid-state infrared-to-visible upconversion sensitized by colloidal nanocrystals. Nat. Photon. 10, 31–34. 10.1038/nphoton.2015.226

[B55] Yan Y. Crisp R. Gu J. Chernomordik B. Pach G. Marshall A. (2017). Multiple exciton generation for photoelectrochemical hydrogen evolution reactions with quantum yields exceeding 100%. Nat. Energy 2:17052 10.1038/nenergy.2017.52

[B56] Yang H. B. Miao J. Hung S. F. Huo F. Chen H. M. Liu B. (2014). Stable quantum dot photoelectrolysis cell for unassisted visible light solar water splitting. ACS Nano 8, 10403–10413. 10.1021/nn503751s 25268880

[B57] Yu W. W. Qu L. Guo W. Peng X. (2003). Experimental determination of the extinction coefficient of CdTe, CdSe, and CdS nanocrystals. Chem. Mater. 15, 854–2860. 10.1021/cm034081k

[B58] Yu X. An X. Shavel A. Ibáñez M. Cabot A. (2014b). The effect of the Ga content on the photocatalytic hydrogen evolution of CuIn_1_-_x_Ga_x_S_2_ nanocrystals. J. Mater. Chem. A 2, 12317–12322. 10.1039/C4TA01315H

[B59] Yu X. Shavel A. An X. Luo Z. Ibáñez M. Cabot A. (2014a). Cu_2_ZnSnS_4_-Pt and Cu_2_ZnSnS_4_-Au heterostructured nanoparticles for photocatalytic water splitting and pollutant degradation. J. Am. Chem. Soc. 136, 9236–9239. 10.1021/ja502076b 24946131

[B60] Zamkov M. (2017). Solar hydrogen generation: exceeding 100% efficiency. Nat. Energy 2:17072 10.1038/nenergy.2017.72

[B61] Zhou Y. Hu W. Ludwig J. Huang J. (2017). Exceptionally robust CuInS_2_/ZnS nanoparticles as single component photocatalysts for H2 evolution. J. Phys. Chem. C 121, 19031–19035. 10.1021/acs.jpcc.7b05241

[B62] Zhu H. Song N. Lv H. Hill C. L. Lian T. (2012). Near unity quantum yield of light-driven redox mediator reduction and efficient H_2_ generation using colloidal nanorod heterostructures. J. Am. Chem. Soc. 134, 11701–11708. 10.1021/ja303698e 22721499

[B63] Zhukovskyi M. Tongying P. Yashan H. Wang Y. Kuno M. (2015). Efficient photocatalytic hydrogen generation from Ni nanoparticle decorated CdS nanosheets. ACS Catal. 5, 6615–6623. 10.1021/acscatal.5b01812

